# Comparative analysis of proteomics and transcriptomics reveals novel mechanism underlying the antibacterial activity and immune-enhancing properties of horse milk

**DOI:** 10.3389/fnut.2025.1512669

**Published:** 2025-03-11

**Authors:** Xueshan Chen, Kawuli Gulbahar, Haiyan Ding, Changhong Nie, Xiaoli Gao

**Affiliations:** ^1^School of Pharmacy, Xinjiang Medical University, Xinjiang, China; ^2^Engineering Research Center of Xinjiang and Central Asian Medicine Resources, Ministry of Education, Xinjiang Medical University, Xinjiang, China

**Keywords:** horse milk, camel milk, goat milk, cow milk, proteomics, transcriptomics, antimicrobial activity, immune induction

## Abstract

**Background:**

Horse milk is a highly valuable organic food that is a promising alternative to cow milk, exhibiting plenty of healthy and immune benefits to human. However, identification of proteins associated human wellness and underlying molecular mechanism in horse milk remain unclear.

**Methodology:**

Label-free mass spectrometry-based protein quantification technology was employed to investigate protein composition of animal milk, including cow, goat, camel and horse milk. Prokaryotic expression and disk diffusion assay were applied to acquire and evaluate *in vitro* antimicrobial activity of candidate proteins. RAW264.7 macrophage model cell line was used to validate effect of proteins on cytotoxicity, apoptosis and immune induction. ROS probe detected cell ROS change and RT-qPCR verified expression of immune response genes induced by proteins. Microscopy was used to observe the effects of protein on the morphological characteristics of bacteria, further transcriptome analysis was performed to investigate transcriptional changes of bacteria induced by candidate proteins.

**Results:**

A total of 1,335 proteins was identified in cow, goat, camel and horse milk. GO enrichment analysis showed that the proteins related to protein degradation were highly expressed in horse milk compared to other three types of milk, contributing to easier assimilation and palatability. KEGG analysis showed that horse milk contained abundant antimicrobial associated proteins relevant to pathogenic bacterial resistance, leading to the decreased risk of pathogenic diseases. A higher accumulation of proteins associated with caffeine metabolism, amino acid biosynthesis, and glycolysis/gluconeogenesis in horse milk contributes to its distinctive flavor. Notably, highly expressed proteins in horse milk were closely linked to immune signaling pathways, functioning as immune modulators. Importantly, we identified four highly expressed antimicrobial associated proteins in horse milk including LPO, B2M, CD14 and PGL, among them, PGL functioned dually by *in vitro* antibacterial activity and immune activation. Further transcriptome analysis demonstrated that PGL exerted significant transcriptional changes to bacteria. Enrichment analysis showed PGL could inhibit growth of *P. aeruginosa* and *E. coli* by repressing the biosynthesis of secondary metabolites.

**Conclusion:**

Comparative proteomics revealed immune enhancement and nutrient composition of horse milk compared to cow, goat and camel milk. Identification of PGL showed antibacterial activity and potential medicinal value.

## Introduction

Milk is a unique substance secreted by the mammary glands of mammals to feed their offspring, and is the perfect food for survival and development after birth (1). Besides human breastmilk, the human habit of consuming milk derived from animal like cow and sheep survived thousands of years and early documented at the Neolithic Age (2). To date, the global milk production of dairy livestock has increased from 522 million tons to 937 million tons from 1987 to 2022, reaching an increase of about 80% (3). Despite the cow milk still had the predominant occupation in milk production, approximately 82% (4, 5), increasing demands for different nutrient compositions in dairy products also facilitated the exploration of other animal derived milk like camel, buffalo, sheep and horse (6–9). Therefore, the researchers take an interest in finding out the value of nutrient and economic in other alternative milk products in current studies.

Horse milk is a promising and valuable dietary resource of dairy products. The inhabitants lived in central Asia and parts of Europe like Holland and Balkan region that consumed horse milk as daily food (10). The nutrient composition and proportions of horse milk are similar with those of human breast milk, including the small proteins (*β*-lactoglobulins and *α*-lactoalbumin), lactose, minerals and microelements (11). Horse milk has an elevated lactose content, excellent palatability, and promotes intestinal calcium absorption, which may contribute to mineralization in children (12). In terms of levels of protein and inorganic content, horse’s milk has a kidney load comparable to that of human milk, implying its economic prospects for infant food (13). The activity and variety of prebiotics and probiotics in horse milk are potentially beneficial for infants and children with cow’s milk protein allergies (CMPA) and intolerances to multiple food ingredients (9, 14). Notably, horse milk was fermented with bacteria and yeasts, forming alcoholic beverages, also called qymyz or koumiss, with unique flavor and taste which shows an appealing trend to consumers (15). Therefore, these studies proposed that horse milk could serve as an excellent alternative of breastmilk and beverage with low digestive burden (16).

Growing evidence now indicated that moderate consumption of horse milk provides significant health benefits across different stages of life (17). The health benefits of horse milk are largely attributed to its higher concentrations of bioactive proteins, such as lactotransferrin (LTF), peptidoglycan recognition protein (PGRP1), and whey acidic protein (WAP), compared to milk from other species, including goat, cow, and buffalo (12). For instance, the replenishment of horse milk could enhance the glycogen level in liver and muscle contributing to the improvement of exercise-induced fatigue (13). Regular consumption of horse milk may help adults lower the risk of chronic diseases, including metabolic syndrome (18), cardiovascular disease (19), and type II diabetes (20). Especially in the elderly, it could enhance cognitive function, preserve skeletal muscle quality, and reduce the risk of frailty and sarcopenia (21). Besides, animal milk can secret milk-derived exosomes (MDEs) containing multiple bioactive molecules such as proteins, microRNAs, and lipids, contributing to intercellular communication and potential immunomodulatory effects (22–24). Sedykh et al. (25) identified several bioactive components like CD81, CD63 receptors, beta-lactoglobulin and lactadherin in horse milk, which function immune contributions to human. Despite such nutritional evidence emphasized the importance of horse milk consumption, limited processing technologies, unknown bioactive components and low production remain in economic promotion of horse milk.

Over the past years, substantial advances have been made in composition determination of various food stuff that is beneficial to human health by proteomic strategy. Proteomic technologies ensure the large-scale and in-depth investigation of proteins, in particular, the determination of potential bioactive substance in complex biological materials by means of a high-throughput manner (26). Renzone et al. (27) detected the quality of advanced glycation end-products (AGE) in various infant formula milk by proteomics analysis which contributed to the risk assessment of infant formula milk. Proteomics analysis of Australian camel milk in different seasons elucidated that summer camel milk contained more abundant accumulation of whey proteins resulting in high nutritional value and easier assimilation (28). Hitherto, few studies have systematically identified bioactive protein components in horse milk. In this study, we used the label-free protein identification approach to quantify and assess difference in proteomes among horse milk, cow milk, goat milk and camel milk. We aimed to investigate major bioactive components and characterized chemical properties of horse milk compared to other types of animal milk. These proteins could be exploited as nutritional marks to highlight the value of horse milk and avail of horse milk promotion. Further identification of antimicrobial associated proteins in horse milk illustrated their immune contributions on inhibitory effect for multiple pathogens and enhancement of host immunity. These findings provide a valuable insight to horse milk protein constitution and immune benefits for further promotion of horse milk.

## Materials and methods

### Sample collection and preparation

Milk samples were collected from Horse (*Equus caballus*), cow (*Bos taurus*), goat (*Capra hircus*), and camel (*Camelus bactrianus*) at 2 weeks after parturition after 2 weeks. Lactating horses were 3 years old, cows were 2 years old, goats were 3 years old and camels were 5 years old, which were selected from a local farm in Nanshan region in Xinjiang. All animals were maintained under standardized dietary and environmental conditions, following established husbandry guidelines. The animals were housed in controlled environments with consistent access to water, regulated temperature, and standardized management practices to reduce external influences on milk composition. Each milk sample was a mixture of milk from three animal individuals and each type of milk sample contained three replicates. A total of 200 mL milk samples was centrifuged at 11,000 r/min for 20 min at 4°C and the upper fat part was carefully discarded. And then, rest solution was freeze-dried into powder by Freeze-Drying Digital Unit (MODULYOD-230) and stored at −80°C, preparing for further analysis.

### Sample preparation for label-free proteomic quantification

A total of 5 mL of SDT cracking solution (sodium dodecyl sulfate, 4% v/v, SDS, dithiothreitol, DTT and Tris–HCl, 100 mM, pH = 7.6) was added to samples of milk from four groups, boiling water bath for 5 min. Then, samples were centrifuged at 14,000 g for 15 min, and supernatant was taken and stored at −80°C for proteome analysis. The protein quantification was detected using BCA method. Take 200 μg protein solution of each sample, add DTT to the final concentration of 100 mM, then boil in water for 5 min, cool to room temperature. Then the solution samples were mixed with 200 μL UA buffer (urea, 8 M, Tris–HCl, 100 mM, pH = 8.5) then transferred to the ultrafiltration centrifuge tube for centrifugation at 14,000 g for 15 min (repeat this step once), and the filtrate was discarded. Subsequently, 100 μL IAA buffer (100 mM IAA in UA) was added to the solution and shake at 600 rpm for 1 min, then the samples were kept at 28°C for 30 min before centrifugation at 14,000 g for 15 min. After centrifugation, 100 μL UA buffer was added, and the samples were centrifuged at 14,000 g for 15 min, and the procedure was repeated twice. In total, 100 μL 25 mM NH_4_HCO_3_ solution was added, centrifuged at 14,000 g for 15 min, and the procedure was repeated twice. Then, 40 μL Trypsin buffer (4 μg Trypsin in 40 μL 100 mM NH_4_HCO_3_) was added into each sample, shake at 600 rpm for 1 min, then the samples were kept at 37°C for 16–18 h. All samples were taken into another collection tube, and centrifuged at 14,000 g for 15 min; Then 40 μL 25 mM NH_4_HCO_3_ was added and centrifuged at 14,000 g for 15 min to collect filtrate. C_18_ Cartridge was used to desalinate the peptide. After lyophilization, the peptide was redissolved with 40 μL 0.1% formic acid solution, and the peptide was quantified with optical density at 280 (OD_280_).

### Protein identification by LC–MS/MS

According to the quantitative results, 2 μg enzymolysis products were taken for LC–MS/MS analysis (Q-Exactive). HPLC system Easy nLC was used for separation. Solution Buffer A is 0.1% formic acid, and solution B is 0.1% formic acid acetonitrile aqueous solution (acetonitrile is 84%). The column was balanced with 95% solution A. The samples were loaded into the Thermo Scientific EASY Column (2 cm*100 μm 5 μm-C_18_), and then analyzed on a Thermo Scientific EASY Column (75 μm*100 mm 3 μm-C_18_) at a flow rate of 300 nL/min. The solution gradient is as follows: 0–110 min, linear gradient of liquid B ranges from 0 to 55%; 110–115 min, the linear gradient of liquid B increased from 55 to 100%; 115 to 120 min, liquid B was maintained at 100%. The peptides were separated by chromatography and analyzed by MASS spectrometry using Q-Exactive Mass spectrometer (Thermo Scientific). Detection method: positive ion; Scanning range of parent ion: 300–1,800 m/z; Primary mass spectrometry resolution: 70,000 at 200 m/z; The peptide fragments was collected according to the following methods: 20 fragments were collected after each Full scan (MS2 Scan), and the resolution of MS was 17,500 at 200 m/z. Microscans: 1, Isolation Window: 2 m/z, Maximum IT: 60 ms, MS2 Activation Type:HCD, Normalized Collision Energy: 27 eV, Dynamic Exclusion: 60.0 s, Underfillratio: 0.1%.

### Data processing and enrichment analyses

The resulting MS/MS data were processed using MaxQuant search engine (vs 1.6.3.3) (29). Tandem mass spectra were searched against the UniProt database concatenated with reverse decoy database. Trypsin/P was specified as cleavage enzyme allowing up to 2 max missing cleavages. The mass tolerance for precursor ions was set as 20 ppm in First search and 6 ppm in Main search, and the mass tolerance was 20 ppm. Carbamidomethyl on Cys was specified as fixed modification and acetylation modification and oxidation on Met were specified as variable modifications.

For protein quantification, label-free quantification (LFQ) was used. The fundamental bioinformatics analyses, including PCA, Pearson correlation and volcano plot analysis, which were conducted using an online cloud-based platform.^1^ Statistical analysis of protein differences was performed using a paired t-test in Perseus (version 1.6.14.0). A significance threshold of *p* < 0.05 and a |Log_2_FoldChange| > 1.0 were applied to identify significantly differentially expressed proteins. KEGG pathway and Gene Ontology (GO) enrichment analyses were conducted to classify the differentially expressed proteins. Fisher’s exact test was used to assess the enrichment of differentially expressed proteins in each category. GO terms and KEGG pathways with *p*-value <0.05 were considered significant.

### Transcriptome analysis

The testing samples were delivered to Allwegene (Nanjing, China) for RNA isolation, and raw data processing was performed using the Allwegene online cloud platform.^2^ For alignment, the clean reads were mapped to the reference genome using the HISAT2 aligner (version 2.2.1). The resulting aligned reads were then normalized using the RSEM (version 1.3.1) tool to account for gene length and sequencing depth biases. Differential expression analysis was performed using the DESeq2 R package (version 1.10.1). DESeq2 performs differential expression analysis by fitting a generalized linear model (GLM) based on a negative binomial distribution. Normalization of read counts was performed using DESeq2 built-in method, which accounts for library size differences between samples. The resulting *p*-values were adjusted using the Benjamini-Hochberg method to control the false discovery rate (FDR). Genes with an adjusted *p*-value <0.05 were considered significantly differentially expressed.

Gene Ontology (GO) analysis was performed using the GOseq R package (version 3.21), which corrects for gene length biases. GO terms were categorized into Molecular Function (MF), Biological Process (BP), and Cellular Component (CC). KEGG pathway analysis was carried out using KOBAS 3.0 software to test the statistical enrichment of differentially expressed genes (DEGs) in KEGG pathways. GO terms and KEGG pathways with a corrected *p*-value <0.05 were considered significantly enriched.

### Prokaryotic expression and protein purification

For prokaryotic expression assay, the coding sequences of PGL, CD14, B2M and LPO were fused with glutathione S-transferase (GST) tag in pGEX-4 T-1 plasmid (kept in local lab, sequence was abstracted from SnapGene 7.1.0) with double-digested by the BamH I and EcoR I sites. Then recombinant plasmids of four proteins were transformed into *Escherichia coli* Rosetta Gami 2 and the transformants were chosen to produce GST-fusion proteins. Recombinant protein expression was induced with 0.4 mM isopropyl-*β*-D-1-thiogalactopyranoside (IPTG) and continuous cultivation in incubator at 18°C, 160 rpm. After 16 h, *E. coli* cells were collected and cleaned in suspension buffer (10 mM Tromethamine-HCl, pH 7.4, 50 mM NaCl). Samples of the *E. coli* suspension culture was pulverized with ultrasound for 20 min and then centrifugated at 12,000 g, 1 h, 4°C. The collected supernatant was purified with GST resin (Sangon, C600912) at 4°C for 1 h in an overhead shaker. The supernatant was then discarded, and the remaining beads were rinsed with glutathione buffer three times before collection. Then sodium dodecyl sulfate polyacrylamide gel electrophoresis (SDS-PAGE) was used to identify whether the target proteins were expressed. Purified recombinant proteins were added to SDS-PAGE loading buffer, boiled, and centrifuged. The supernatants were subjected to PAGE, and the proteins were transferred to polyvinylidene difluoride membranes. The membranes were incubated with the indicated antibodies in protocols.

### Antimicrobial activity *in vitro*

A bacterial suspension with 1 × 10^4^ CFU/mL was evenly spread onto agar plates (100 μL per plate) and allowed to adsorb for 5 min. Recombinant proteins were lyophilized by Freeze-Drying Digital Unit (MODULYOD-230) and reconstituted to a final concentration of 200 mg/L. Protein concentration was determined using the Bradford protein assay (BSA) (Sangon biotech, Shanghai). A BSA standard curve was prepared by serial dilution of a BSA stock solution, and the absorbance was measured at 595 nm using a spectrophotometer. Ampicillin sodium (50 μg/mL) served as the positive control and GST protein served as the negative control. Sterile paper discs (0.5 cm diameter) were loaded with 12 μL of either the protein solution or the control. The discs were aseptically placed onto the agar surface using flame-sterilized tweezers, ensuring firm contact with the medium. Each bacterial strain was tested in triplicate, with three independent replicates. Plates were incubated at 37°C for 16–24 h, and the diameter of inhibition zones was measured using a ruler. The inhibitory effectiveness was evaluated by subtracting the paper disc diameter from the inhibition zone diameter.

### RNA extraction and RT-qPCR assays

The RNA of RAW264.7 cell was extracted using the TRIzol method (Invitrogen, CA, United States) and treated with RNase-free DNase I (Takara, Kusatsu, Japan). Total RNA (2 μg) was reverse-transcribed to cDNA in a 20 μL reaction mixture using a SPARKscript II RT Plus.

Kit (SparkJade, Jinan, China). The qPCR instructions were conducted as described by Jia et al. (30). The primers used for qPCR are listed in Supplementary Table S1. The internal control was used as GAPDH (Accession number: NM_001256799.3).

### Cell culture, apoptosis staining, and detection assays

RAW264.7 cells were obtained from China Center for Type Culture Collection (CCTCC, Wuhan, China) and grown in Dulbecco’s modified Eagle’s medium (DMEM) using 12-well plates and treated with 500 μM H_2_O_2_ for 1 h to construct oxidative-inflammation model cell. Then, the model cells were treated with recombinant proteins of PGL, LPO, CD14 and B2M with 10 mg/mL for 1 h. The cell viability was determined using MTT. RAW264.7 cells were incubated with H_2_O_2_ (500 μM) for 1 h, containing recombinant proteins with 10 mg/mL for 1 h. Hoechst33342, PI and DCFH-DA (10lM) staining were performed following Ma et al. (31). All cell culture experiments were performed at 37°C and 5% CO_2_ conditions. Apoptosis feature was analyzed by ImageJ. Each determination contained three biological replicates and was repeated three times.

### Statistical analysis

Statistical analysis was performed using GraphPad Prism 8.0.1 (GraphPad Software, San Diego, CA, United States). Data are presented as mean ± standard deviation (SD). A one-way analysis of variance (ANOVA) followed by Tukey’s *post-hoc* test was used to determine statistical significance. *p*-value <0.05 was considered statistically significant.

## Results

### Proteomic patterns of horse milk separated with other three kinds of mammal milk

**Figure 1:**
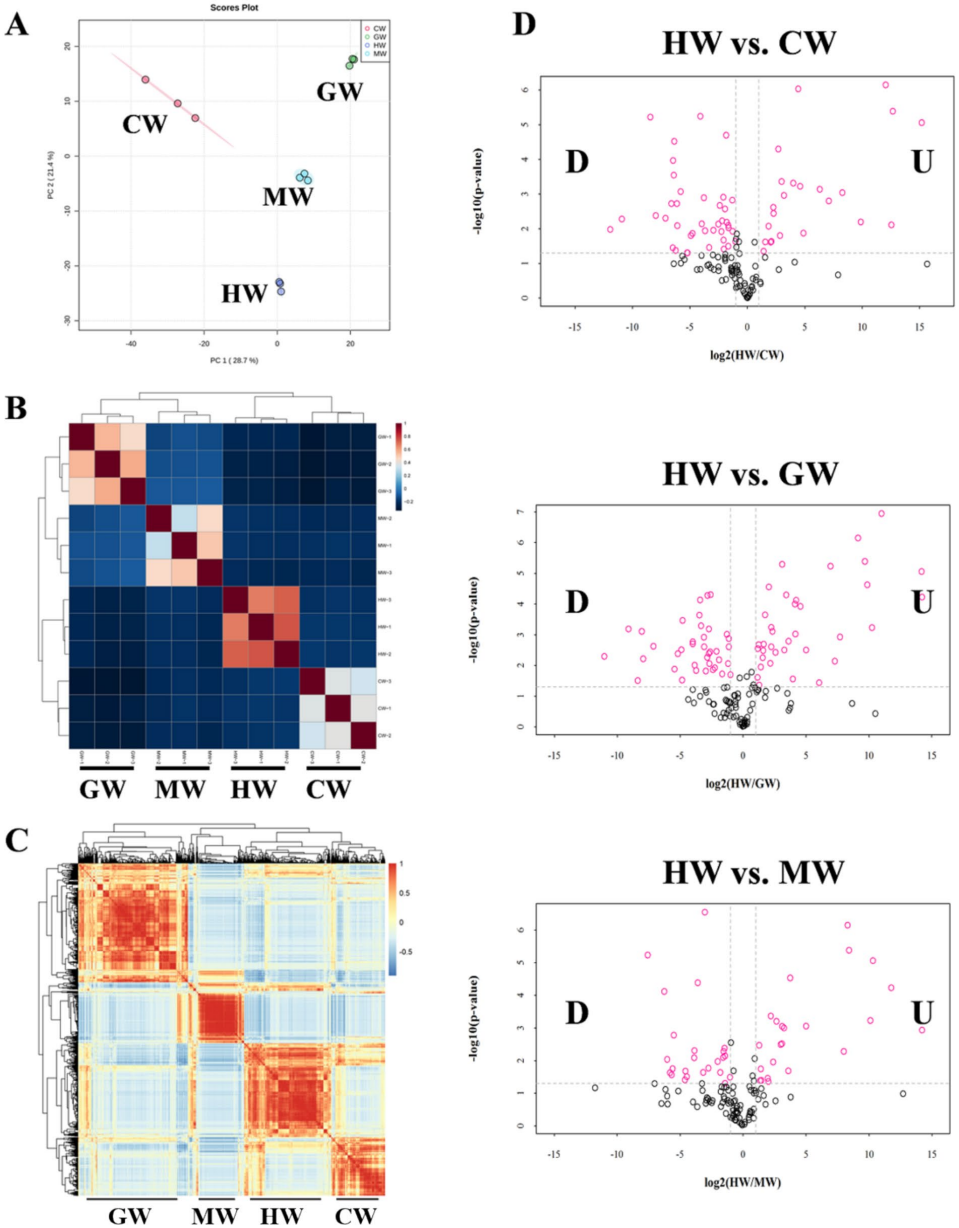
Overview of proteomic profiles from four organisms. **(A)** Score plot of principal component analysis (PCA) of the proteome datasets. Each plots represented a sample in experiment. **(B)** Hierarchical clustering displayed the correlation among all samples. The samples from same organisms closely clustered together at one clade and clearly separated with other three groups of samples. **(C)** Correlation analysis among all proteins in the proteomic profiles. Red and blue represented the high and low correlation relationship between two proteins, respectively. **(D)** Volcano plot (*p*-value versus fold change ratio) displayed the significantly differentially expressed proteins in each comparison (HW vs. CW, HW vs. GW and HW vs. MW). Red dots are significant at *p*-value <0.05.

Horse milk is characterized as abundant content of proteins, including caseins, whey proteins and lactoferrin, which is beneficial for human health and immunity (26). However, the detailed information about constitution and distribution of these proteins or other beneficial proteins among several mammal milk remains unclear. We then performed an in-depth proteome analysis on milk from healthy horse, cow, goat and camel (HW: horse milk in wellness; MW: Cow milk in wellness; GW: Goat milk in wellness, CW: camel milk in wellness) to illustrate the difference of protein composition among them. We first developed the principal component analysis of proteomic profiles in four groups. The result showed that distinct clustering of the four groups (CW, GW, HW, and MW), with PC1 and PC2 explaining 28.7 and 21.4% of the total variance, respectively, suggesting their variable proteomic compositions (Figure 1A). Intriguingly, we noticed that HW group was distinguished from other three groups with markedly separated distribution (Figure 1A). Consistently, Pearson correlation analysis displayed similar results that all samples from same group clustered together, and formed four clades including HW, MW, GW, and CW, demonstrating the credibility of data and differences among samples of four types of milk (Figure 1B). Further detailed representative proteins were mapped in correlation analysis and showed that there were four sets of proteins among these samples, which reached a consensus with the number of experimental groups (Figures 1B,C). The correlation analysis confirmed the significantly different compositions of proteomic profiles among four types of animal milk which in favor of next work.

Subsequently, we set threshold *p*-value <0.05 and |Log_2_foldchange| > 1.0 to identified differentially expressed proteins. In HW vs. CW comparison, 34 proteins were identified to upregulate in HW, while 89 proteins were downregulated in HW (Figure 1D). In parallel, there are 53 upregulated and 68 downregulated proteins in HW vs. GW comparison (Figure 1D). Then, compared with MW, the expression levels of 41 proteins were higher in HW, whereas the levels of 73 proteins were lower in HW (Figure 1D). And these proteins will be recognized as focus for further analysis to explain the advantages of horse milk in consumption.

### The protein component and function analysis between horse milk and cow milk

**Figure 2:**
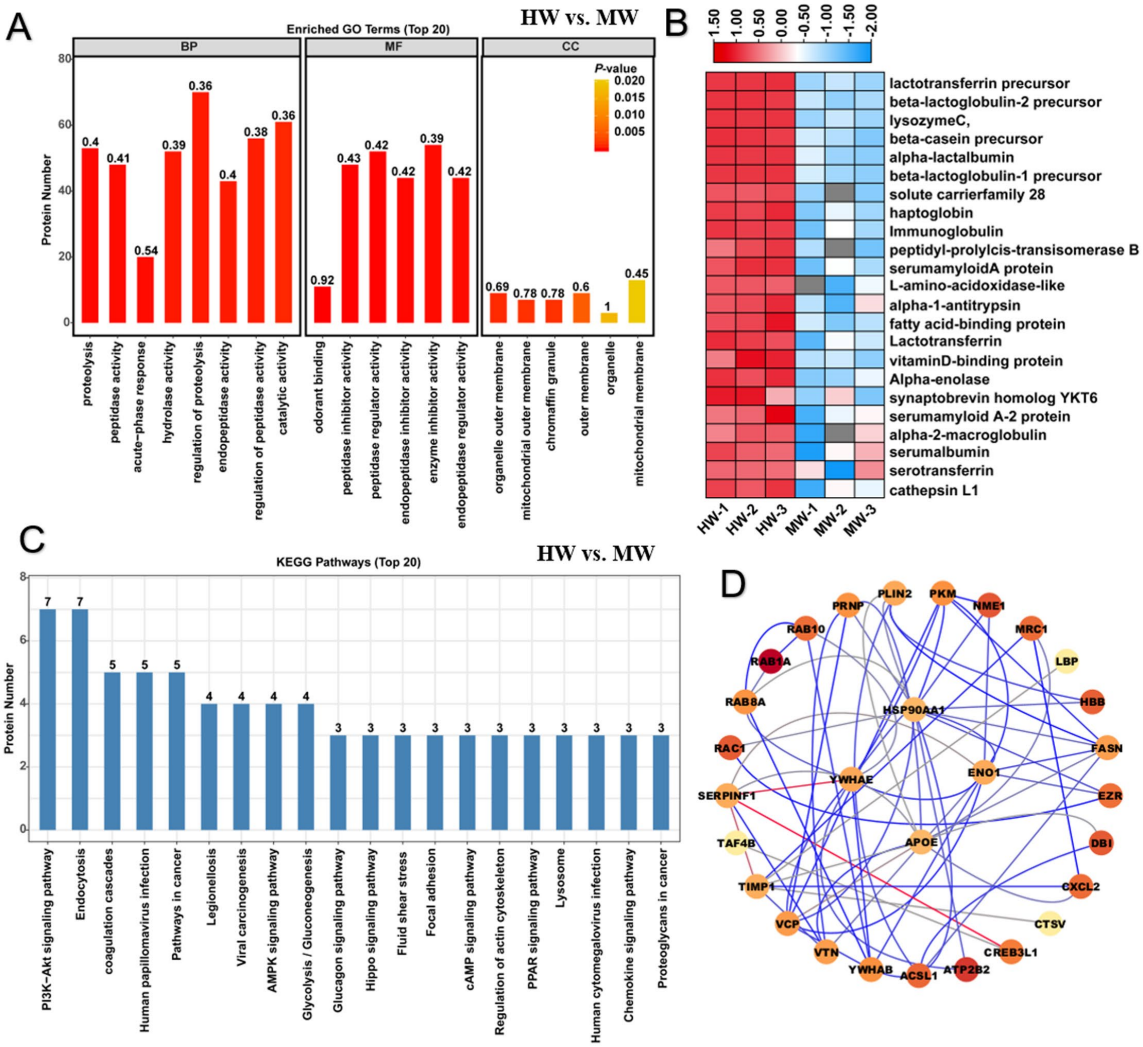
GO and KEGG enrichment analysis illustrated difference of protein function between horse milk and cow milk. **(A)** GO enrichment analysis on differentially expressed proteins in HW vs. MW. Three main terms represented by molecular function, cellular component and biological process were shown the histogram. **(B)** Heatmap displayed the relative expression levels of proteins in HW and MW. The up- and downregulated proteins were shown in red and blue, respectively. The scale represented the normalized expression values of each protein. **(C)** KEGG enrichment analysis on differentially expressed proteins in HW vs. MW. **(D)** The construction of protein–protein interaction network for differentially expressed proteins in HW vs. MW.

Horse milk is considered to be a promising alternative for cow milk, to investigate the difference of protein constitution between them, we performed Gene ontology analysis on the differentially expressed proteins in HW vs. MW. GO analysis provides a dynamically updating controlled vocabulary set to describe genes and gene product attributes in an organism. Previous identification of 69 up-regulated and 72 down-regulated proteins (Figure 1D) were mapped into GO enrichment analysis. The result showed that these proteins were mainly enriched in 121 GO terms containing 96 BP terms, 9 CC terms and 16 MF terms. For terms of BP, we found that these proteins involving in protein proteolysis, peptidase activity, acute-phase response, hydrolase activity, regulation of proteolysis, regulation of endopeptidase activity, regulation of peptidase activity and catalytic activity were significantly enriched (*p* < 0.05) (Figure 2A). This implied that the proteins relevant to proteolysis activity were more active in horse milk compared to cow milk, which caused the difference of digestive absorption between them. For terms of MF, we found these proteins were predominantly gathered in odorant binding, peptidase inhibitor activity, peptidase regulator activity, endopeptidase inhibitor activity, enzyme inhibitor activity, endopeptidase regulator activity, vitamin D binding, serine-type endopeptidase inhibitor activity, molecular function regulator, transporter activity, enzyme regulator activity, calcidiol binding, pheromone binding, D_3_ vitamins binding, vitamin binding and lipid binding (*p*< 0.05) (Figure 2A). These results suggested that proteins associated protein degradation and activity varied between horse milk and MM, suggesting the advantages of horse milk may contained more proteins related to degradation. For terms of CC, we found these proteins were mainly enriched in organelle outer membrane, chromaffin granule, mitochondrial outer membrane, outer membrane, extracellular membrane-bounded organelle, mitochondrial membrane, mitochondrial envelope, apical dendrite and mitochondrial part (*p* < 0.05) (Figure 2A). The results suggested the location of these proteins which function proteolysis and enzyme activity. We thus proposed that HW was rich in proteins functioning active proteolysis, degradation and catalysis compared to MW, which may contribute to absorbable and palatable feature of horse milk. As expected, expression pattern of these proteins with up-regulation (foldchange>2 in HW vs. MW) was further abstracted in proteome file and depicted in heatmap (Figure 2B), showing most of proteins were from whey proteins including lactotransferrin, lysozyme C, lactoglobulin, immunoglobulin, macroglobulin, serum albumin and serotransferrin, which showed significant increase in content compared to MW. These results supported that horse milk contained more whey protein components that were easier to digest and absorb, and these whey proteins also showed beneficial goodness to human health and immunity.

Further KEGG enrichment pathway analysis was used to determine protein distribution in metabolic pathway. We noticed that several DEPs were significantly enriched in immune signaling pathways or disease resistance pathways (Figure 2C). Especially seven significantly enriched metabolic pathways including PI3K-Akt signaling pathway, MAPK signaling pathway, shigellosis, salmonella infection, *Escherichia coli* infection, legionellosis and endocytosis overrepresented the metabolic direction of most proteins (*p* < 0.05) (Figure 2C; Supplementary Table S2). These results suggested that horse milk contained more abundant proteins potentially related to immune regulation that may contribute to trigger immune signaling pathways or reduce the risk for contaminates by such pathogenic microbes in horse milk. Additionally, these proteins were further used to construct protein–protein interaction network, the result showed that four hub proteins like HSP90AA1, YWHAE, APOE, and ENO1 functioned pivotal roles in regulation of immune regulatory network in horse milk (Figure 2D). Studies have shown that these proteins functioned as modulators in the regulation of NF-κB and MPK signaling pathways, contributing to host defense against pathogen infections (32–34). Therefore, our findings demonstrated that HW had advantages on elevated accumulation of whey proteins and immune-related proteins for human health and immunity compared to MW.

### The protein component and function analysis between horse milk and goat milk

**Figure 3:**
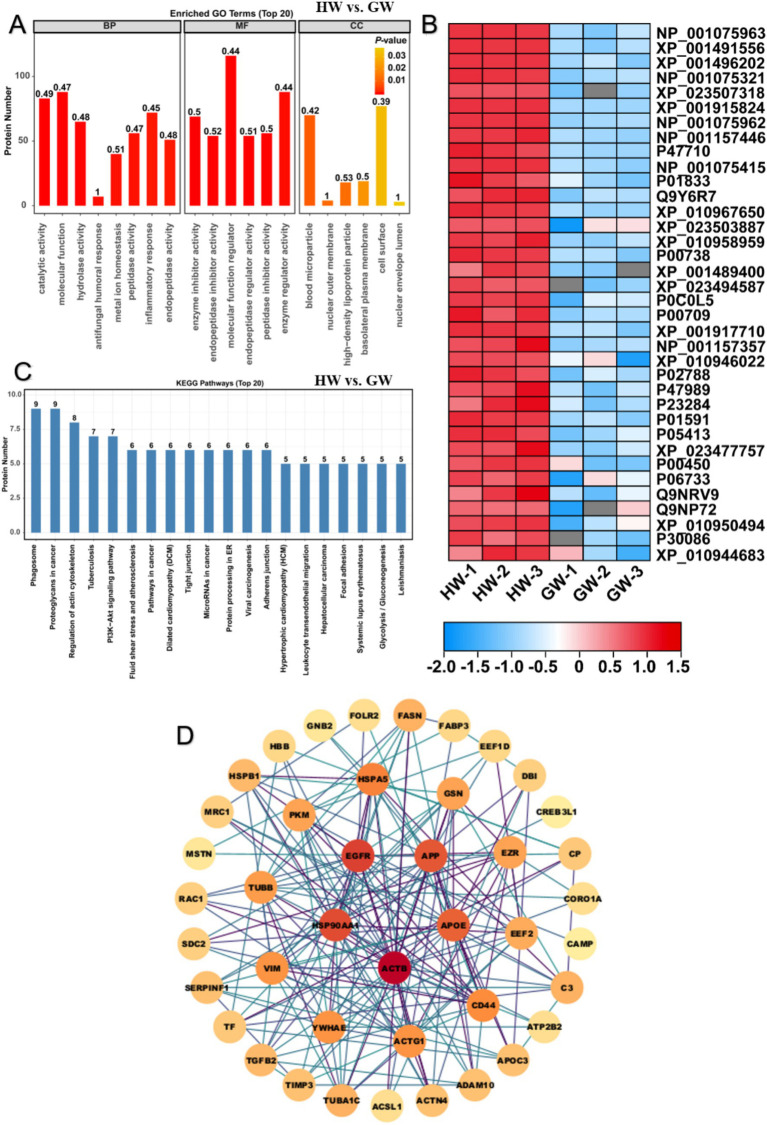
GO and KEGG enrichment analysis illustrated difference of protein function between horse milk and goat milk. **(A)** GO enrichment analysis on differentially expressed proteins in HW vs. GW. Three main terms represented by molecular function, cellular component and biological process were shown the histogram. **(B)** Heatmap displayed the relative expression levels of proteins in HW and GW. The up- and downregulated proteins were shown in red and blue, respectively. The scale represented the normalized expression values of each protein. **(C)** KEGG enrichment analysis on differentially expressed proteins in HW vs. GW. **(D)** the construction of protein–protein interaction network for differentially expressed proteins in HW vs. GW.

In the comparison of HW vs. GW, 108 up-regulated proteins and 114 down-regulated DEPs were into GO enrichment analysis and found that these proteins were mainly enriched in 275 GO terms containing 230 BP terms, 9 CC terms and 36 MF terms. For terms of BP, we found that these proteins were remarkably clustered in BP term of the negative regulation of catalytic activity (*p* < 0.01, FDR < 0.05) (Figure 3A). Besides, we also noticed that proteins related to negative regulation of catalytic activity, negative regulation of molecular function and inflammatory response were significantly enriched (Figure 3A). The results implied that significant difference of protein components between both types of milk proteins was the variation of catalytic activity which may account for different nutrition constitution and flavor between them. For terms of MF, we found these proteins were predominantly gathered in enzyme inhibitor activity, endopeptidase inhibitor activity, molecular function regulator, endopeptidase regulator activity, peptidase inhibitor activity and enzyme regulator activity (*p* < 0.05, FDR < 0.1) (Figure 3A). The result showed that proteins associated protein degradation and activity were significantly enriched in HW compared to GW, suggesting more active protein degradation and digestion events occurred in HW. Indeed, low molecular weight protein in milk composition was readily available to assimilation for human. We thus proposed their difference of absorption should be taken into consideration in further assessment between them. For terms of CC, we found these proteins were predominantly enriched in blood microparticle, nuclear outer membrane, high-density lipoprotein particle, basolateral plasma membrane, cell surface, nuclear envelope lumen, HFE-transferrin receptor complex, nuclear chromatin and phagocytic cup (*p* < 0.05) (Figure 3A). The results suggested the location of these proteins which function regulator or enzyme activity. Expression of these proteins were further depicted in heatmap (Figure 3B), illustrating that most of whey protein components were up-regulated in HW compared to GW, leading to the higher benefits for human health and immunity.

Based on previous result, 108 up-regulated proteins and 114 down-regulated proteins were further mapped into KEGG enrichment pathway analysis in the comparison of HW vs. GW. The results showed that the DEPs linked in such immune signaling pathways representing main function of them, including PPAR signaling pathway, HIF-1 signaling pathway, ferroptosis, ECM-receptor interaction and complement and coagulation cascades were significantly enriched (Supplementary Table S3). PPAR (peroxisome proliferators-activated receptor) signaling pathway partook in the physiological processes of lipid metabolism, cell proliferation and differentiation (35). HIF-1 (hypoxia- inducible factor-1) signaling pathways promote the adaption to low oxygen tension in cells and organisms resulting in the transcriptional induction of a series of genes that participate in angiogenesis, iron metabolism, glucose metabolism, and cell proliferation/survival (36). It has been reported that the higher accumulation of proteins relevant to PPAR, HIF-1 signaling pathways and ferroptosis enhanced the risk of inflammatory bowel disease (37, 38). Thus, these results suggested that HW could avoid to stimulate organic inflammatory response and reduce the risk of pathogenic microbes compared to GW. Meanwhile, most of proteins were gathered in such metabolic pathways, including phagosome, proteoglycans in cancer, regulation of actin cytoskeleton, tuberculosis pathogenic *E. coli* infection and MAPK signaling pathway (Figure 3C). We noticed that these enriched proteins probably functioned as antibodies associated with the pathogenic bacterium species like shigellosis, salmonella and *E. coli* that mainly caused bowel diseases. it was documented that horse milk exhibited the inhibitory effect to *Salmonella Typhimurium* by suppressing the virulence gene expression (*hilA* and *ssrB*2) (39). Moreover, donkey belonged to Equus genus, was found to contained many antimicrobial factors by proteomic analysis, implying the antimicrobial activity of donkey’s milk (40). We thus speculated that HW may contain some immune related proteins that exerted the positive therapy effect to multiple disease compared to GM. Further construction of protein interaction network showed five hub immune-related proteins such as HSP90AA1, ACTB, APOE, APP, and EGFR functioned crucial roles in manipulation of host immune response in horse milk (Figure 3D), which may take part in prevention of pathogenic microbes or immune trigger in horse milk.

### The protein component and function analysis between horse milk and camel milk

**Figure 4:**
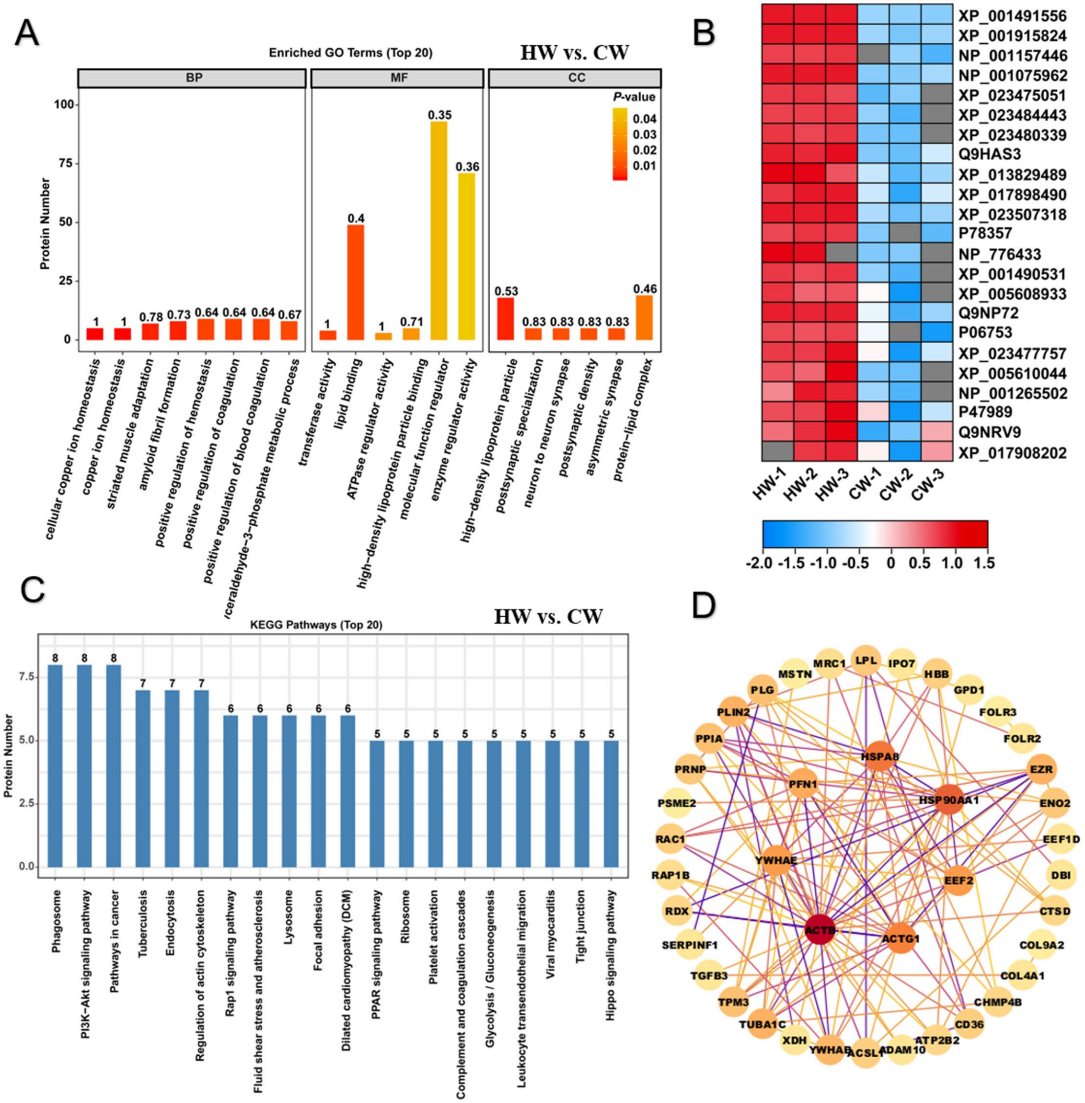
GO and KEGG enrichment analysis illustrated difference of protein function between horse milk and camel milk. **(A)** GO enrichment analysis on differentially expressed proteins in HW vs. CW. Three main terms represented by molecular function, cellular component and biological process were shown the histogram. **(B)** Heatmap displayed the relative expression levels of proteins in HW and CW. The up- and downregulated proteins were shown in red and blue, respectively. The scale represented the normalized expression values of each protein. **(C)** KEGG enrichment analysis on differentially expressed proteins in HW vs. CW. **(D)** the construction of protein–protein interaction network for differentially expressed proteins in HW vs. CW.

For differentially expressed proteins in HW vs. CW comparison, GO functional enrichment analysis was performed on all 34 upregulated and 89 downregulated proteins (Figure 4A). The results cover a wide range of terms relevant to molecular functions (MF), biological processes (BP) and cellular components (CC). There are 84 terms with *p* < 0.05 were significantly enriched against these 123 differentially expressed proteins (Figure 3A). For terms of cellular component (CC), these proteins mainly performed functions at high-density lipoprotein particle, postsynaptic specialization, neuron to neuron synapse, postsynaptic density, asymmetric synapse, protein-lipid complex, plasma lipoprotein particle, lipoprotein particle, preribosome, membrane microdomain and membrane raft (Figure 4A). And these proteins mainly performed seven kinds of functions, including transferase activity, transferring aldehyde or ketonic groups, lipid binding, ATPase regulator activity, high-density lipoprotein particle binding, molecular function regulator, enzyme regulator activity and ATPase binding, implying their importance in nutritional support and bioavailability of nutrients in horse milk (Figure 4A). Additionally, for biological process category (BP), 66 related terms were significantly overrepresented among these proteins, including cellular copper ion, homeostasis, copper ion homeostasis, striated muscle adaptation, amyloid fibril formation, positive regulation of hemostasis, positive regulation of coagulation, positive regulation of blood coagulation, glyceraldehyde-3-phosphate metabolic process, glucose 6-phosphate metabolic process, pentose-phosphate shunt, muscle atrophy, striated muscle atrophy, sterol transport, cholesterol transport, regulation of lipid catabolic process, positive regulation of lipid catabolic process, regulation of cytokine production involved in immune response, protein kinase A signaling, muscle adaptation and negative regulation of fibrinolysis (Figure 4A). Previous studies mentioned the benefits of horse milk for contributing to homeostasis and bone development, and we noticed that the enrichment of several proteins was closely associated with positive regulation of hemostasis, muscle atrophy and striated muscle atrophy, implying that horse milk exhibited a potential therapy effect to bone disease (Figure 4A). Meanwhile, various proteins involved in primary metabolisms were enriched, such as regulation of lipid catabolic process, glyceraldehyde-3-phosphate and cholesterol transport (Figure 4A). Heatmap illustrated expression level of these associated proteins showing most of them belonged to whey protein components with significant up-regulation in HW compared to CW (Figure 4B). These results suggested the advantages of horse milk in improving immunity and chemicals which could be used as energy and nutrient for human, compared with CW.

Further pathway enrichment analysis on these proteins showed that they were mapped onto 144 KEGG pathway, with 5 pathways were significantly enriched against these proteins, including phagosome, PI3K-Akt signaling pathway, pathways in cancer, rap1 signaling pathway, MAPK signaling pathway and pathogenic *Escherichia coli* infection (Figure 4C; Supplementary Table S4). Further construction of protein interaction network showed potential interacted and regulatory relationship among them, we noticed six hub proteins including HSP90AA1, EEF2, ACTG1, ACTB, YWHAE, PFN1, and HSPAB functioned essential roles in regulation of these proteins in immune response (Figure 4D). These proteins may act as triggers or antigens in response to pathogenic microbes’ invasion.

### Increased accumulation of immune-related proteins in horse milk conferred more healthy benefits

**Figure 5:**
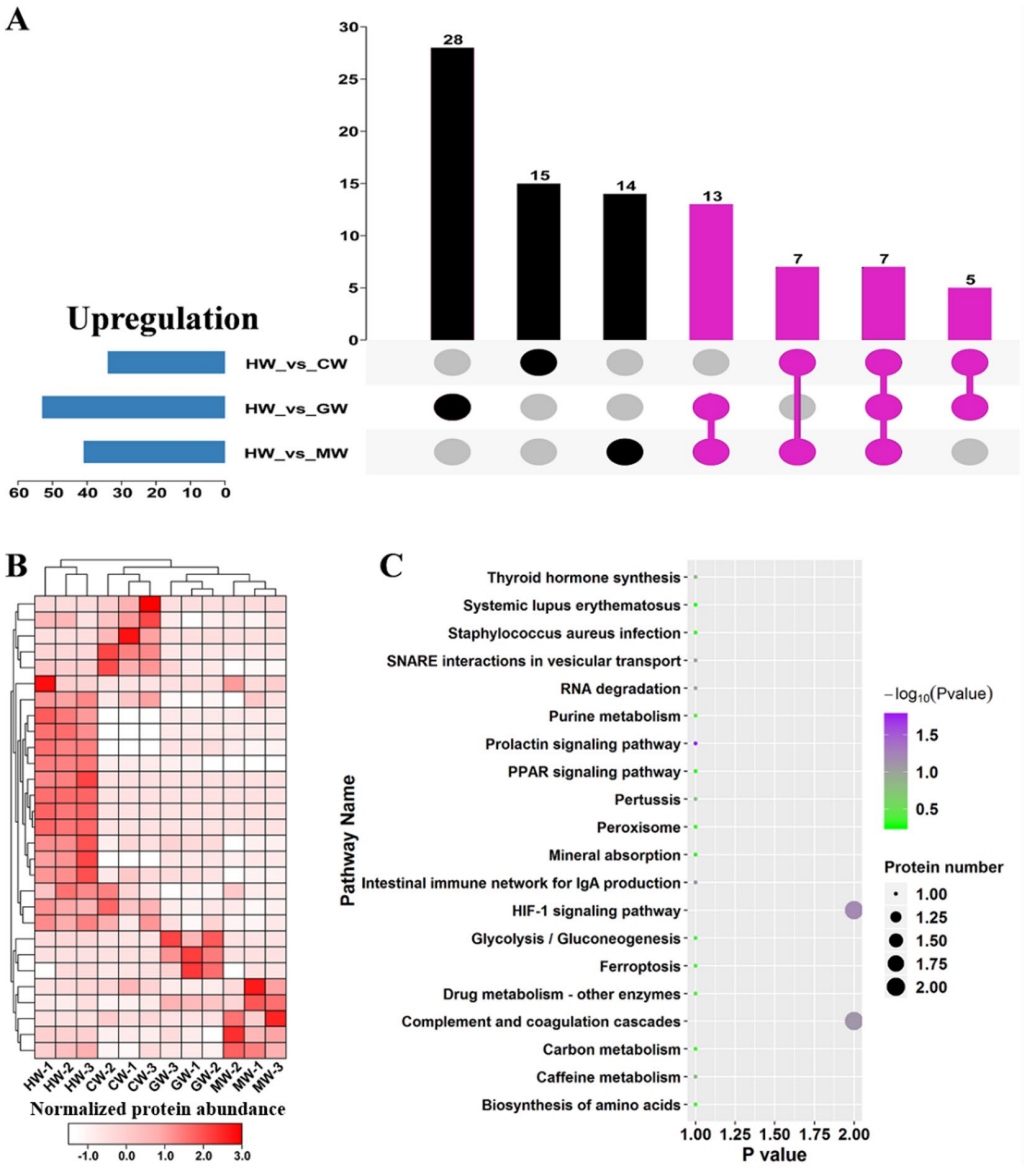
identification and function enrichment analysis on hub upregulated proteins in HW. **(A)** Upset diagrams representing the overlap of significantly upregulated proteins in HW vs. CW, HW vs. GW and HW vs. MW pairwise comparisons. The hub upregulated proteins of HW were labeled by purple. **(B)** Clustering analysis of hub upregulated proteins in HW. The proteins with relative high expression level were shown in red, whereas the relative low expression level was shown in white. **(C)** KEGG pathway classification of hub upregulated proteins.

To identified the hub proteins that highly expressed in HW compared to other three organisms, we analyzed all upregulated proteins in HW vs. CW, HW vs. GW and HW vs. MW comparisons (Figure 5A). As shown in Figure 5A, we identified 32 candidate proteins shared in at least two comparisons. Remarkably, in total 7 proteins were shared in three comparisons, suggesting their levels were significantly higher in HW than other three organisms (Figures 5A,B). Then, we analyzed the expression levels of all 32 proteins among all samples, and identified 15 proteins that highly expressed in HW than other three organisms (Figure 5B). These 15 proteins were recognized as hub proteins which contributed to the advantages of horse milk. Especially four whey protein components including *α*-lactalbumin (XP_001915824), lysozyme C (XP_001491556), *β*-lactoglobulin-1 precursor (NP_001075962) and lactotransferrin precursor (NP_001157446) showed significant accumulation in HW compared to other three types of milk.

The pathway enrichment analysis is the most intuitive way to understand the advantages of horse milk, thus KEGG pathway enrichment analysis was employed to reveal the functional roles of these 15 hub proteins (Figure 5C). The results showed the overrepresentation of 20 pathways among these 15 hub proteins associated with HW, including prolactin signaling pathway, HIF-1 signaling pathway, intestinal immune network for IgA production, Complement and coagulation cascades, RNA degradation, SNARE interactions in vesicular transport, caffeine metabolism, thyroid hormone synthesis, pertussis and drug metabolism (Figure 5C). IgA has been implicated in functioning center roles in the maintain of normal development and suppressing harmful bacterial microbes (9). We noted various pathways relevant to immunity were identified, including HIF-1 signaling pathway, Intestinal immune network for IgA production and complement and coagulation cascades (Figure 5C), suggesting that usage of horse milk could improve human immunity. Additionally, various metabolisms relevant to beneficial chemicals were found to be active in HW, like amino acids and glycolysis/gluconeogenesis (Figure 5C), indicating that horse milk is rich in these related proteins.

### The identification of antimicrobial associated proteins in horse milk

To further investigate the molecular function of these seven shared proteins with high expression in horse milk, we examined the expression profiles and listed them in Table 1, including LPO, B2M, NAMLAA, CD14, TLR2, PG4, and LTFp. Notably, we found that the expression of LPO, B2M, NAMLAA, and CD14 was significantly increased in three pairwise comparisons, suggesting the four candidate proteins may confer strong antimicrobial activity in horse milk.

**Table 1:** The expression profiles of unique antimicrobial associated proteins in HW.

NCBI ID	Annotation	Abbreviation	Log_2_FC (HW/CW)	Log_2_FC (HW/GW)	Log_2_FC (HW/MW)
XP_014596904.2	Lactoperoxidase	LPO	1.33**	1.91**	1.42**
XP_005602652.1	Beta-2-microglobulin	B2M	1.21**	1.51**	1.32**
XP_023481087.1	N-acetylmuramoyl-L-alanine amidase	NAMLAA (PGL)	1.25**	1.1*	1.21**
XP_023490830.1	Monocyte differentiation antigen CD14	CD14	1.52**	1.08*	1.18**
XP_023481835.1	Toll-like receptor 2	TLR2	0.88	1.01*	1.21**
XP_023494587.1	Platelet glycoprotein 4	PG4	0.74	0.87	1.19**
NP_001157446.1	Lactotransferrin precursor	LTFp	0.84	1.01**	1.34**

*P < 0.05, **p < 0.01.

**Figure 6:**
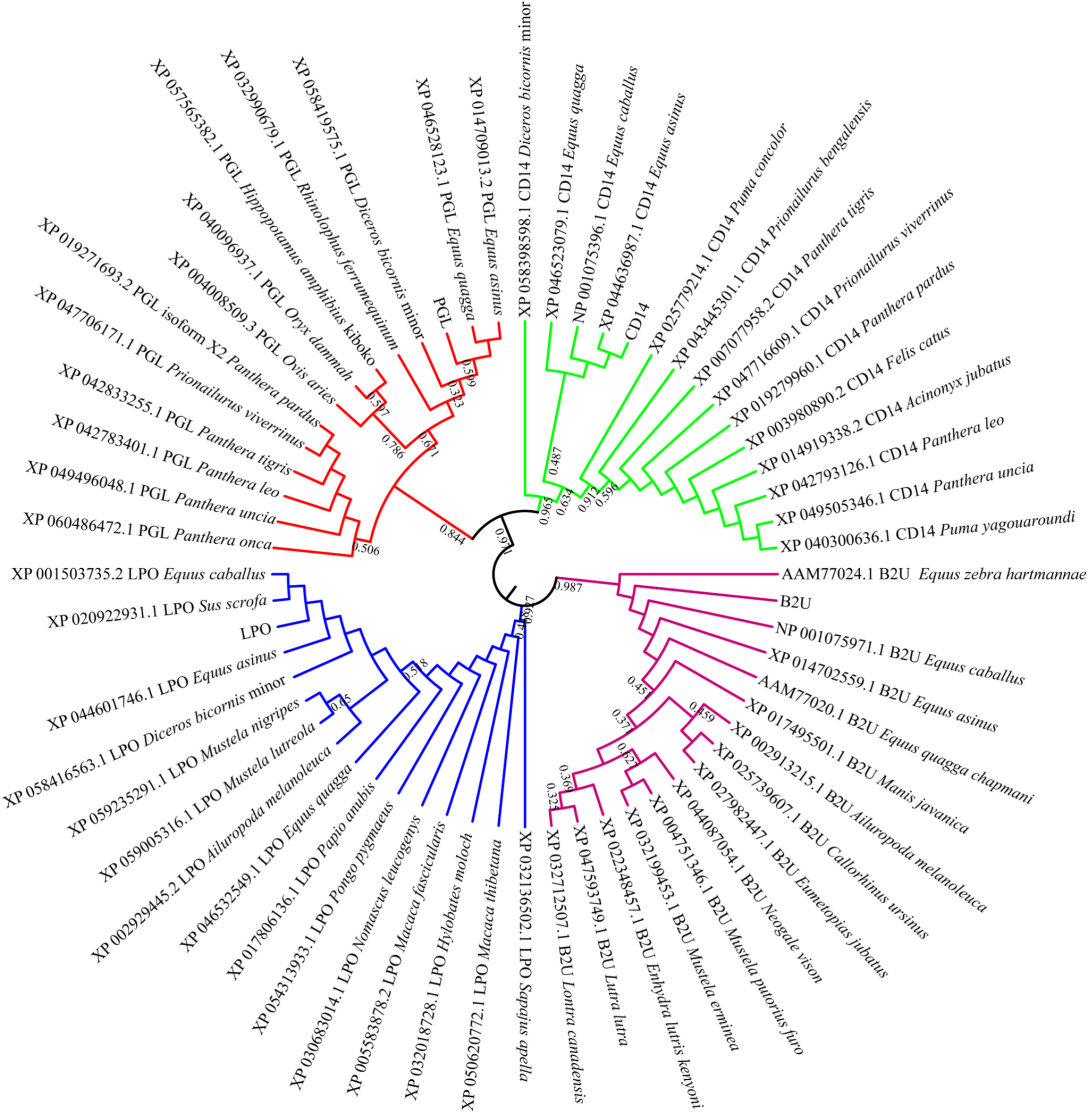
Phylogenetic tree of candidate proteins in horse milk.

To explore the sequence characterization of these candidate proteins, a phylogenetic tree was constructed to evaluate their evolutionary relationships. As shown in Figure 6, we found that LPO was clustered with its homologous protein derived from *Equus caballus*, *Sus scrofa*, *Equus asinus*, and *Diceros bicornis*. NAMLAA (PGL) had an aggregation with proteins coming from *Equus quagga* and *Equus asinus*. CD14 had a high sequence conservation in multiple horse species including *Equus caballus*, *Equus asinus*, and *Equus quagga*. And Equus-derived B2M proteins were also closely gathered in one clade. In common, the candidate proteins were sequence-conservative in Equus genus, suggesting their conservative molecular function in horse milk.

**Figure 7:**
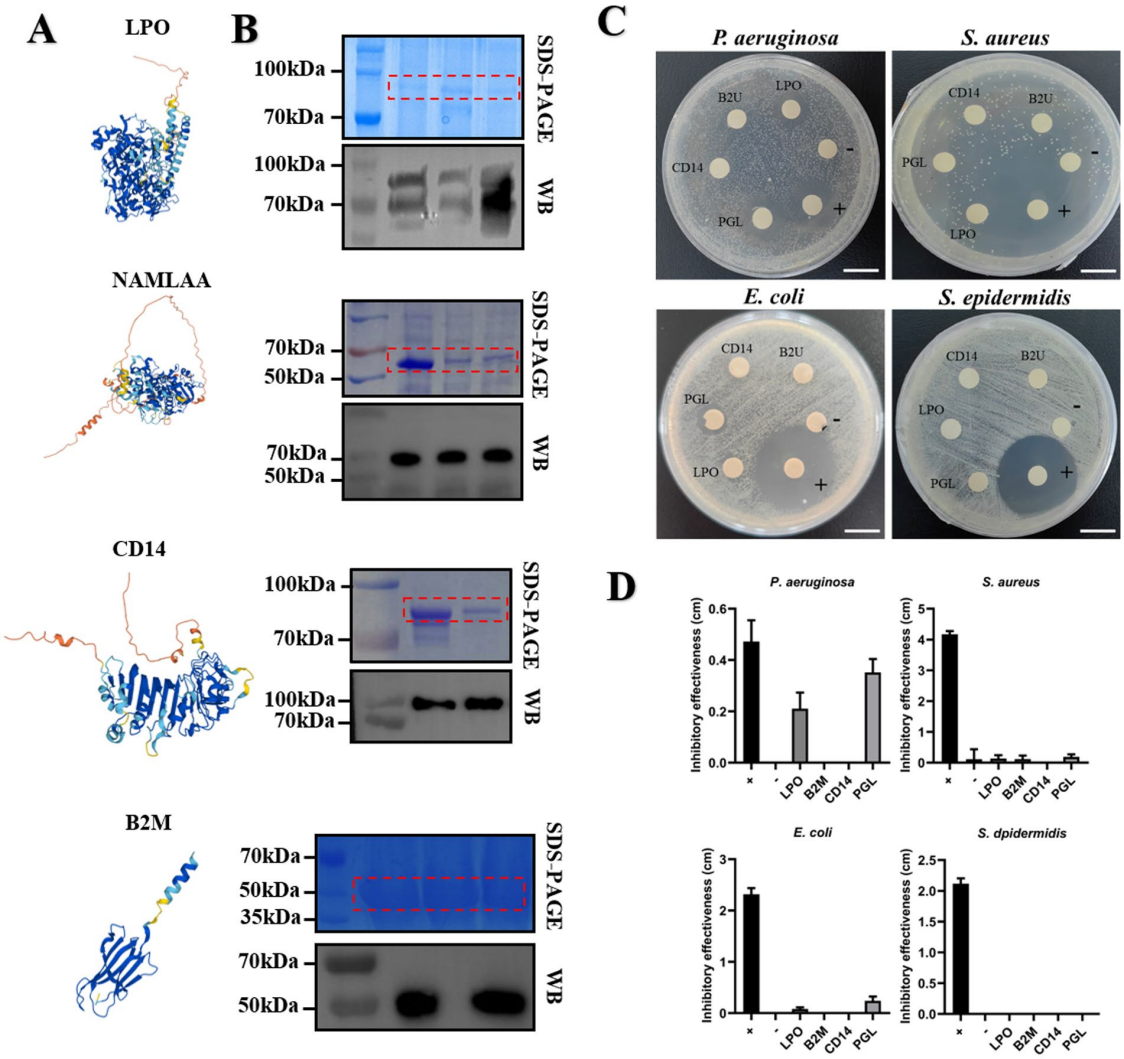
Candidate proteins in HW conferred antimicrobial activity for multiple pathogenetic microorganisms. **(A)** The probable protein structure model predicted by AlphaFold database. **(B)** The authorization of SDS-PAGE and Western Blot for expression of candidate proteins. **(C)** *In vitro* antimicrobial activity assay tested the inhibition effect of candidate proteins responding to multiple microorganisms. The inhibitory effect of these pathogens was evaluated by paper disk method. GST protein was used as negative control and ampicillin sodium with the concentration of 50 μg/mL was used as positive control. Scale bar is 2 cm. **(D)** The assessment of inhibitory effectiveness of candidate proteins. Effectiveness was evaluated by subtracting the paper disc diameter from the inhibition zone diameter.

Based on AlphaFold protein structure database^3^ (41), we predicted probable protein structure of four candidate proteins, depicting them as Figure 7A. Among them, B2M protein showed relatively simple structure, containing a *α*-helix and five *β*-sheets. And these structures also could be spotted in other three proteins, which play a crucial role in maintaining protein conformation stability under various stress conditions, thereby ensuring the proper execution of its biological functions. Studies have shown that B2M played a role in regulating immune responses, antigen presentation, and immune tolerance, especially interaction with defensin protein elevating resistance to multiple pathogens invasion (42). LPO is a peroxidase widely present in mammalian milk, playing a crucial role in antibacterial activity, immune regulation, and oxidative stress control (43). PGL is a class of enzymes involved in bacterial cell wall degradation, contributing to cell wall metabolism, bacteriolysis, and host immune defense (44). CD14 activates innate immunity and enhances downstream antimicrobial components, including LPO and lysozyme, to protect against pathogenic infections (45). We then performed codon optimization of NAMLAA (PGL), CD14, and LPO for next protein purification. The optimized open reading frame (ORF) of candidate proteins was cloned and fused with pGEX-4 T-1 for prokaryotic expression. Further SDS-PAGE and Western Blot assays authorized the successful expression of four candidate proteins (Figure 7B). Purified proteins fused with GST tag were lyophilized to condense at the concentration of 10 mg/mL. We then performed an *in vitro* antimicrobial activity assay to assess the capacity of inhibition against multiple pathogenic microorganisms. In total, four bacterial strains including *Pseudomonas aeruginosa*, *Staphylococcus aureus*, *Escherichia coli*, and *Staphylococcus epidermidis* were chose for testing object. As shown in Figure 7C, exogenous application of PGL and LPO showed marked inhibition to *P. aeruginosa* growth (Figure 7D). In anti-*Escherichia coli* assay, only application of PGL could inhibit pathogen growth (Figures 7C,D). None of the tested antimicrobial proteins exhibited significant inhibitory effects against *S. epidermidis* and *S. aureus* (Figures 7C,D). Overall, our results suggested that the usage of horse milk improved human immunity attributing to abundant accumulation of proteins with direct antimicrobial activity.

### Elevated immunity in horse milk by four antimicrobial proteins

**Figure 8:**
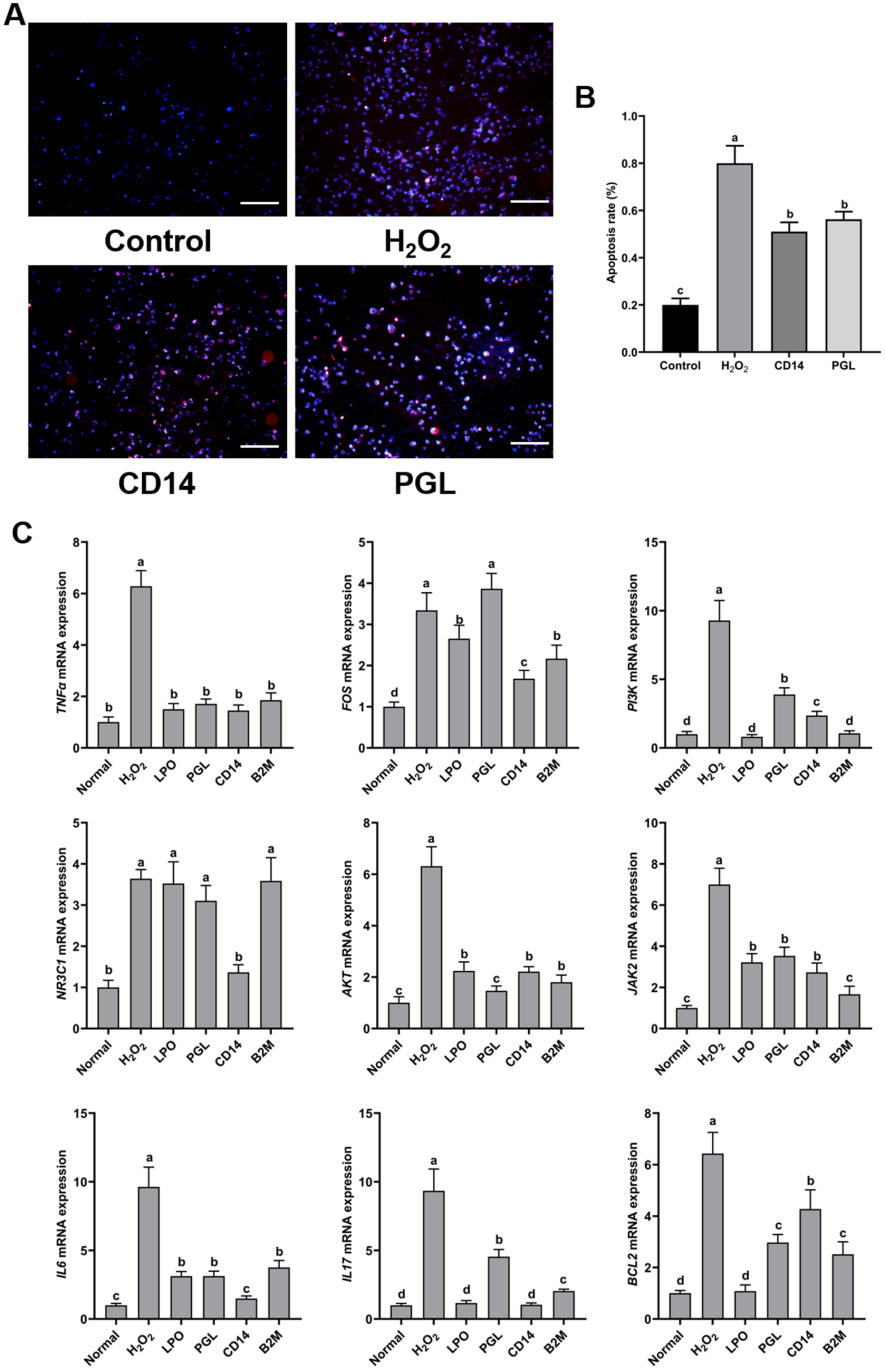
Exogenous application of CD14 and PGL inhibited apoptosis and enhanced host immunity. **(A)** The apoptosis of horse milk treated with PGL and CD14 protein and hydrogen peroxide was observed, scale bar is 50 μM. **(B)** Cell apoptosis statistics between treatments. Different letters represent significant differences between treatments. **(C)** The relative expression levels of immune-related genes in H202, LPO, PGL, CD14, and B2M treated horse milk were detected by RT-qPCR. Normal indicates normalization. The relative expression levels of immune-related genes in LPO, PGL, CD14, and B2M treated horse milk were detected by RT-qPCR, normal indicates normalization. Error bars represent the standard deviation of the means. Different letters represent significant differences between treatments.

To further evaluate the resistant contributions of four antimicrobial protein in immunity, we exogenously treated RAW264.7 cells with four antimicrobial proteins. First, we examined the toxicity of four proteins on RAW264.7 cells. The results showed that exogenous treatment with antibacterial proteins at 5–20 mg/mL did not significantly inhibit cell viability, ensuring that none of the four antibacterial proteins were toxic to RAW264.7 cells (Supplementary Figure S1). H_2_O_2_ is a well-recognized inducer of apoptosis. Our research discovered that pre-treatment with the four antibacterial proteins reduced H_2_O_2_-induced apoptosis in RAW264.7 cells, with CD14 and PGL demonstrating significant inhibitory effects (Supplementary Figure S1). Further electron microscopy observation showed that PGL and CD14 proteins could inhibit H_2_O_2_-induced apoptosis, significantly reducing the apoptosis rate by approximately 25~30% (Figures 8A,B). This supports the notion that PGL and CD14 proteins possess anti-apoptotic activity in RAW264.7 cells. To further investigate the immune effects of the core proteins LPO, PGL, CD14, and B2M on their host, we treated RAW264.7 cells with the core proteins LPO, PGL, CD14, and B2M, along with H_2_O_2_, for 1 h. Samples were collected for RNA extraction and reverse transcription into cDNA. qRT-PCR was then used to measure the relative expression levels of immune-related genes *AKT*, *BCL2*, *FOS*, *IL17*, *IL6*, *JAK2*, *NR3C1*, *PI3K*, and *TNFa* (Figure 8C). These genes were relatively upregulated in RAW264.7 cells treated with H_2_O_2_. Importantly, *CD14* and *PGL* notably induced the upregulation of immune-related genes, excluding *TNFa*, thereby enhancing the immune response in RAW264.7 cells.

### Exogenous treatment of PGL inhibited pathogen growth by interference of secondary metabolism

**Figure 9:**
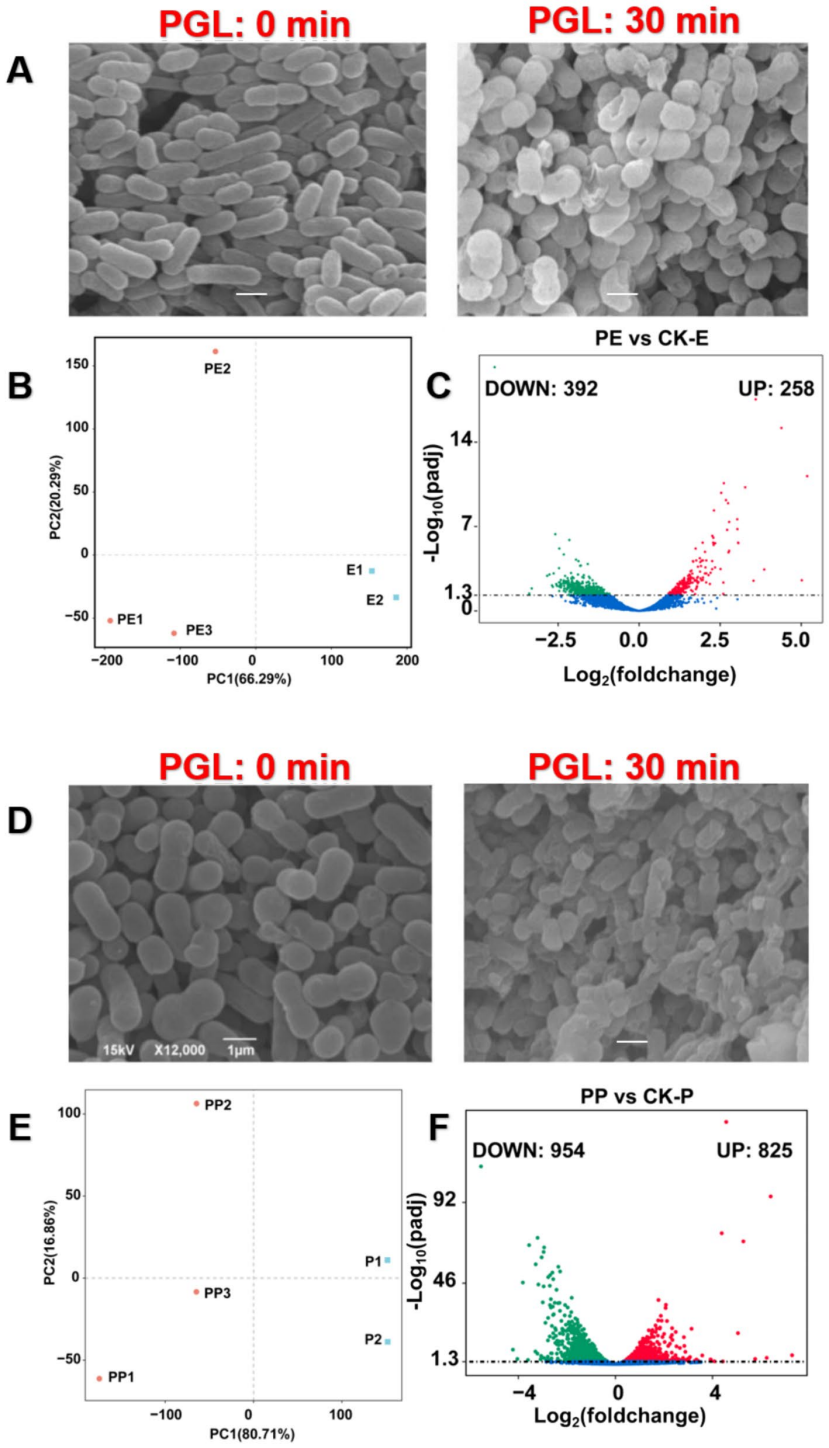
A global view of transcriptome files of *Escherichia coli* and *Pseudomonas aeruginosa* treated by PGL. **(A,D)** Observations of the morphology of *E. coli* and *P. aeruginosa* after 30 min of PGL protein treatment under scanning electron microscopy. The left image shows the normal morphology of normal pathogens (×12,000), while the right image shows the morphology of pathogens (×12,000) after 30 min of protein PGL treatment. Scale bar is 1 μm. **(B,E)** Principal component analysis of transcriptome files of *Escherichia coli* and *Pseudomonas aeruginosa* treated by PGL as well as controls. **(C,F)** Identification of differentially expressed genes (DEGs) in PE vs. CK-E and PP vs. CK-P using volcano plots.

Through scanning electron microscopy, we directly observed that PGL exogenous treatment for 30 min caused damage to the cell membrane structure of *Escherichia coli* and *Pseudomonas aeruginosa* (Figures 9A,D). However, the molecular mechanism by which it induces host immunity is still unclear. Therefore, we continued to explore the transcriptional changes in these two bacteria under PGL treatment by transcriptome analysis. First, PCA analysis revealed significant transcriptional changes in *E. coli* and *P. aeruginosa* after PGL treatment, resulting in 80.71 and 66.29% of transcriptomic differences, respectively (Figures 9B,E). This supports that PGL treatment caused major transcriptional changes in these bacteria. Volcano plots showed that, compared with the control, PGL treatment resulted in 258 upregulated and 392 downregulated differentially expressed genes in *E. coli*, and 825 upregulated and 954 downregulated differentially expressed genes in *P. aeruginosa* (Figures 9C,F). These differentially expressed genes were mapped to specific functional blocks through further GO and KEGG enrichment analysis, reflecting the transcriptional changes induced by PGL in host immunity.

**Figure 10:**
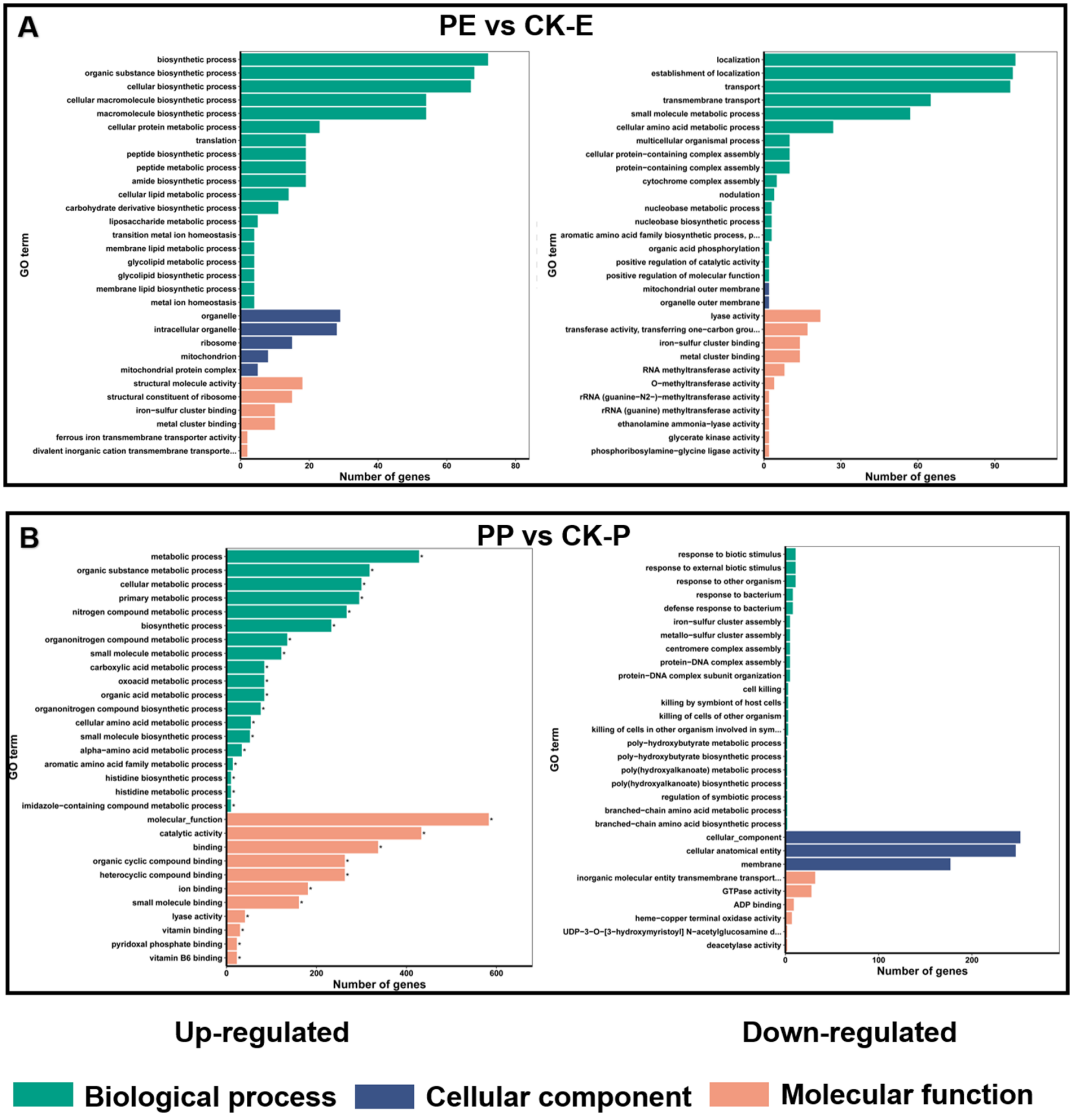
GO enrichment analysis illustrated the functional distribution of differentially expressed genes in PE vs. CK-E and PP vs. CK-P. **(A)** GO enrichment analysis of up-regulated and down-regulated DEGs in PE vs. CK-E. **(B)** GO enrichment analysis of up-regulated and down-regulated DEGs in PP vs. CK-P.

Secondly, GO enrichment analysis indicated that the differentially expressed genes in *E. coli* were mainly distributed in biological process categories, with upregulated genes significantly enriched in intracellular biomolecule synthesis and downregulated genes mainly associated with transmembrane and localization (Figure 10A). These results suggest that PGL may interfere with the synthesis and transport of intracellular biomolecules, thereby affecting the growth of *E. coli*. In *P. aeruginosa*, the upregulated differentially expressed genes were predominantly enriched in cell metabolism and catalytic activity categories, while the downregulated genes were mainly related to intracellular components and membrane components (Figure 10B). This indicates that PGL might disrupt normal catalytic processes, affecting the integrity of intracellular components.

**Figure 11:**
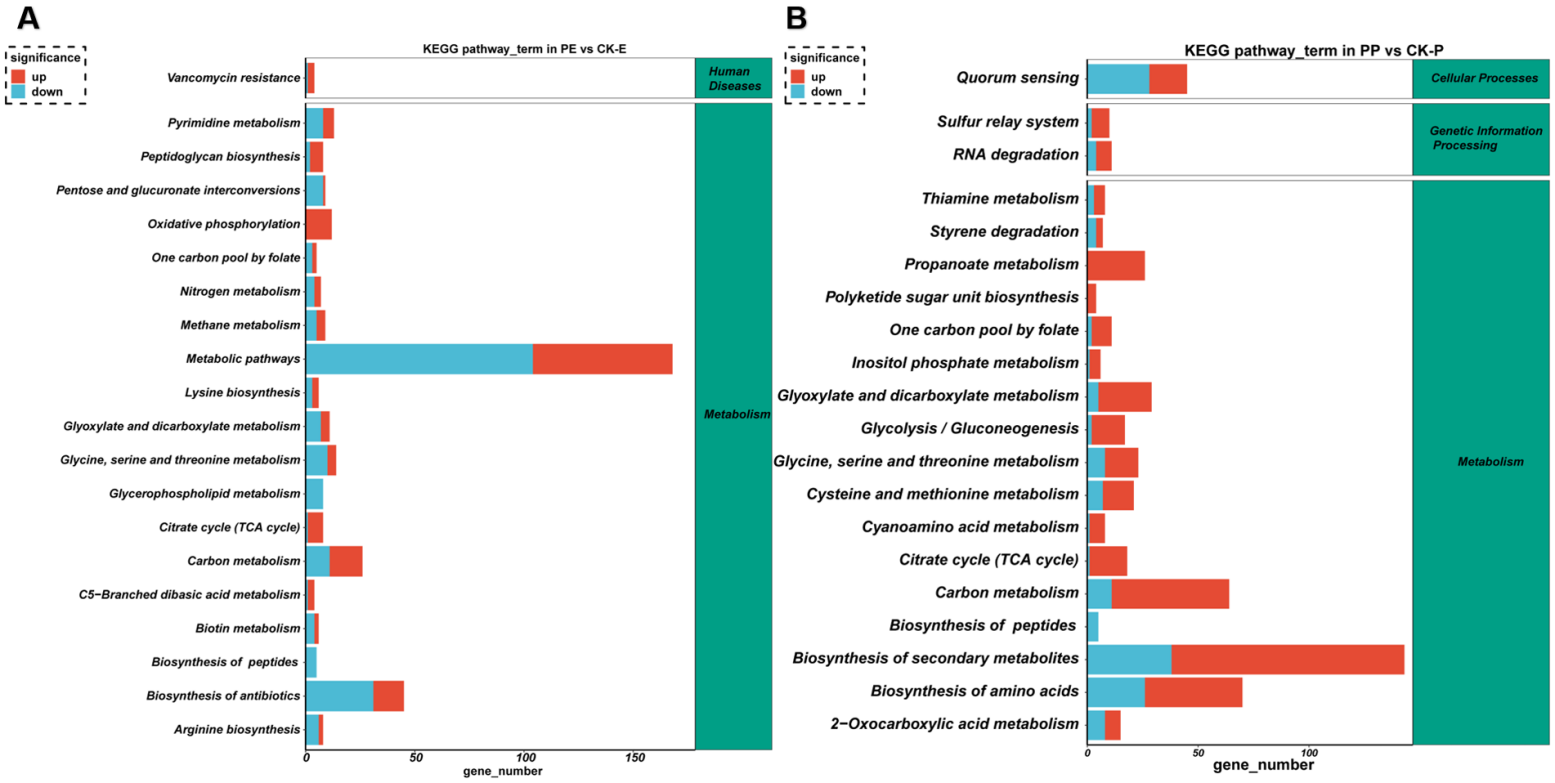
KEGG enrichment analysis illustrated the metabolic pathways of differentially expressed genes in PE vs. CK-E and PP vs. CK-P. **(A)** KEGG enrichment analysis of DEGs in PE vs. CK-E. **(B)** KEGG enrichment analysis of DEGs in PP vs. CK-P.

KEGG analysis revealed that the differentially expressed genes in *E. coli* were primarily enriched in pathways including oxidative phosphorylation, metabolic pathways, carbon metabolism, and biosynthesis of antibiotics. Notably, there was a majority of downregulated genes in pathways related to metabolism and antibody synthesis. These findings indicate that PGL inhibits metabolic processes and antibiotic synthesis in *E. coli*, leading to the suppression of pathogenic bacterial growth (Figure 11A). In *P. aeruginosa*, differentially expressed genes were significantly enriched in pathways related to biosynthesis of secondary metabolite, biosynthesis of amino acids and carbon metabolism, suggesting that PGL exogenous treatment disrupted the normal pathways of secondary metabolite synthesis, leading to inhibition of pathogenic bacterial growth (Figure 11B). In summary, these findings indicate that PGL suppresses the growth of pathogenic bacteria by disrupting the synthesis of secondary metabolites.

## Discussion

Researches on more nutritious and affordable milk source are of paramount importance. This study investigated the changes in protein and metabolites abundance in cow, goat, camel and horse milk by proteomic analysis. We summarized our acquired evidence depicting them as following conclusions. (a) Horse milk varied from other types of milk (cow, goat and camel) in protein composition. (b) Horse milk contained proteins distinguished from others with readily available for proteolysis, absorption and human immunity. (c) The increased content of proteins in horse milk contributed to elevate immune response and resistance to pathogenic disease. (d) The Identification of PGL protein conferred inhibitory effects to *Escherichia coli* and *Pseudomonas aeruginosa* by repressing biosynthesis of secondary metabolites.

Despite the low yield of horse milk because of lactation and longer gestation, its nutritional value was emphasized due to abundant whey protein and microelements (46). Our data verified that whey proteins occupied the most proportion in total proteins of horse milk among these four types of animal milk. Whey proteins were not only readily to digest and absorb, but also contained various amino acids that are essential for human, like lysine (Lys), leucine (Leu), glutamic acid (Glu), and aspartic acid (Asp), which were beneficial to muscle growth, energy production and gut health (47). As a comparison, cow milk contained plentiful casein proteins compared to whey proteins (casein: whey =4: 1), especially A1-*β* casein, which has been reported to be directly associated with milk intolerance (48). Huge demands for cow milk around the world, though there still cannot be neglected the cow milk intolerance. Our data highlighted the advantage of whey protein in horse milk, and proteomic analysis showed that horse milk contained more abundant proteins relevant to proteolysis, degradation and catalysis, suggesting the potential benefits of horse milk in absorption and palatability. Like casein proteins with large molecular weight were uneasy to degrade, which could be decomposed into small casein peptides by caseinase in milk (49). Besides, active proteins related to caffeine metabolism, biosynthesis of amino acids and glycolysis/gluconeogenesis in horse milk suggested potential contributions to flavor and taste of horse milk. Despite these advantages, positioning horse milk as an easily assimilated dietary alternative, several challenges limit its widespread production and consumption. Its lower protein and fat content may limit its nutritional appeal, while high lactose levels pose concerns for lactose-intolerant individuals (50). Production constraints, including low yield and seasonal lactation, restrict large-scale commercialization. Additionally, storage stability, microbial susceptibility, and optimal pasteurization require further investigation (46). Future research should focus on enhancing preservation strategies, refining processing techniques, and exploring its immunomodulatory and antimicrobial potential to expand its applications in health and nutrition.

In recent years, increasing interests has been taken in the immunomodulatory properties of dairy products, especially for immune function and modulating immune responses. Our data suggested a promising application of horse milk on therapy effect to multiple human diseases. Proteomic analysis indicated that proteins relevant to shigellosis, salmonella infection, *E. coli* infection, tuberculosis and thyroid cancer were highly enriched in horse milk compared to other types of milk (cow, goat and camel milk). Intriguingly, many proteins in horse milk were remarkably enriched in immune pathways, such as PI3K-Akt, Rap1, PPAR, and MAPK signaling pathways, which has been emphasized in many documents as essential hub governor to modulate apoptosis, inflammation and immunity events in organic development (51). These results suggested potential immune benefits of horse milk.

The functional characterization of antimicrobial proteins in horse milk provides critical insights into its quality, bioactivity, and unique properties. As an underexplored dairy source, horse milk harbors a diverse repertoire of bioactive proteins with potential roles in host defense, microbial balance, and immune modulation. Our identification of antimicrobial components in horse milk contributed to advances our understanding of horse milk’s functional attributes but also paves the way for its broader application in nutrition and health. Donkey belonged to Equus genus, previous studies demonstrated that donkey’s milk contained many antimicrobial factors with inhibitory effect (40). Our identification of PGL and LPO with conservative evolutionary relationships in Equus genus, which conferred direct antimicrobial activity to inhibit the growth of *P. aeruginosa* and *E. coli*. Consistent with our finding, Guri et al. (39) manifested that some active antibacterial proteins existed in horse milk that resulted in an inhibitory effect to *Salmonella Typhimurium* growth. *β*-defensin in human, well-known for their broad-spectrum antimicrobial activity, are key players in mucosal immunity and pathogen defense (52). It has been reported that the content of lysozyme protein in horse milk was higher than that in cow and human milk, which also implied higher antimicrobial activity in horse milk due to lysozyme capability (53). Besides, we speculated that natural antibacterial activity of candidate proteins could ensure horse milk to maintain a relatively long shelf life and a low rate of pathogenic contamination without excessive processing, awaiting to be further studied.

Despite these findings illustrated the importance of antimicrobial proteins in inhibitory effect, immune activation by them should not be ignored. Enhancement of immune resistance by horse milk diet has been reported in recent years, especially in the treatment of tuberculosis and chronic ulcer (54). In this study, exogenous application of LPO, PGL, CD14, and B2M enhanced host immune response by elevating expression of immune-related genes like *AKT*, *PI3K*, *IL6*, *IL17* and so on, highlighting their contributions to host immunity in horse milk. Among them, we identified that PGL functioned dually by direct antibacterial activity and priming immune signaling in pathogen resistance. Our transcriptome analysis demonstrated that PGL could repress transcription related to key secondary metabolism pathways in both bacterial. These pathways are crucial to produce various metabolites that contribute to the bacterial virulence and resistance mechanisms. The inhibition of these pathways by PGL suggests a disruption to bacteria’s metabolic versatility and ability to adapt to environmental stressors. We reasoned that this disruption may exert profound implications for antimicrobial resistance (AMR), as secondary metabolites are often involved in the production of resistance factors. We proposed that further investigation into the metabolic changes induced by antimicrobial proteins like PGL could lead to innovative therapeutic interventions, providing a promising avenue for combating the growing threat of antimicrobial resistance.

## Conclusion

In this study, we highlight the unique advantages of horse milk in immune enhancement and nutrient composition, demonstrating its potential as a functional food and an alternative for individuals with cow milk allergies. Our identification of proteins (B2M, PGL, CD14, and LPO) in horse milk contributes to human immunity. Among them, PGL function triply by *in vitro* antibacterial activity, immune activation and interference with secondary metabolism in pathogenic bacteria. Our findings insights into comprehension of horse milk compounds and molecular immune mechanism, expecting to provide a theoretical basis for promotion of horse milk. Molecular insights into identification of bioactive compounds in horse milk provide theoretical basis for therapeutic applications, broadening the dietary and medical potential of horse milk.

## Data Availability

The original contributions presented in the study are included in the article and Supplementary material, further inquiries can be directed to the corresponding author.

## Footnotes

^1^ https://www.omicstudio.cn

^2^ http://218.2.224.234:8888

^3^https://alphafold.ebi.ac.uk (Accessed May 28, 2024).
