# Supplementary figures and images for: Comparative analysis of proteomics and transcriptomics reveals novel mechanism underlying the antibacterial activity and immune-enhancing properties of horse milk
Source: Front Nutr. 2025 Mar 11;12:1512669. doi: 10.3389/fnut.2025.1512669 (PMC11932903; doi:10.3389/fnut.2025.1512669)

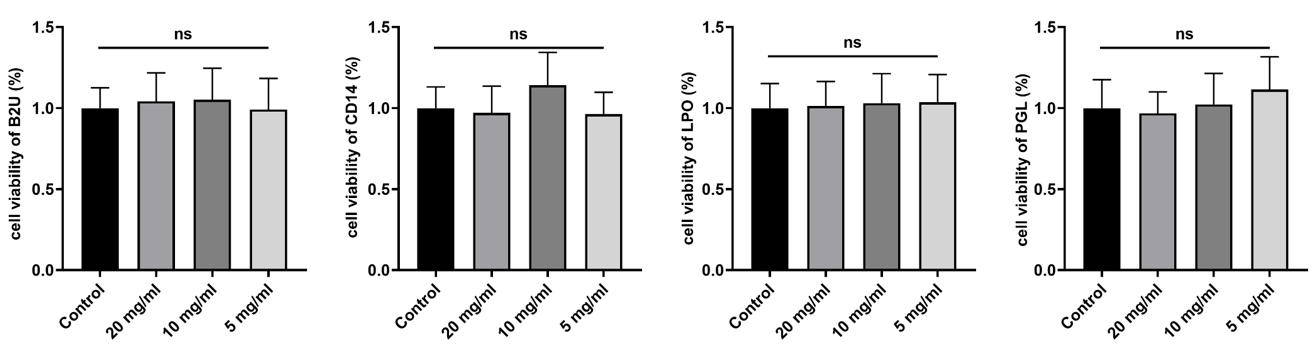

Supplement: Supplementary file 1 [file Data_Sheet_1.zip › Figure S1 The analysis of cell viability in different tested cells exogenous treated by antimicrobial proteins..tif]
